# Primary Bone Leiomyosarcoma in Neurofibromatosis Type 1: Extremely Rare Concurrency

**DOI:** 10.7759/cureus.20811

**Published:** 2021-12-29

**Authors:** Izzeddin J Abualjubain, Muath Mamdouh Mahmod Al-Chalabi, Wan Azman Wan Sulaiman

**Affiliations:** 1 Reconstructive Sciences Unit, Universiti Sains Malaysia (USM), Kota Bharu, MYS; 2 Plastic and Reconstructive Surgery, Universiti Sains Malaysia School of Medical Sciences, Kota Bharu, MYS

**Keywords:** primary bone leiomyosarcoma, leiomyosarcoma, sarcoma, plexiform neurofibroma, neurofibromatosis type 1

## Abstract

Neurofibromatosis type 1 (NF1) is a complex autosomal dominant, multisystem genetic disease affecting about 1 in 3500 individuals. Plexiform neurofibromas represent a rare variant (30%) of NF1 in which the spread of tumor cells along nerve fascicles leads to a diffuse mass of thickened nerve fibers. Affected patients with NF1 have a greater chance of developing soft tissue sarcomas than the general population. Leiomyosarcoma is one of the most frequent soft tissue sarcomas, seldom observed in patients with NF1. Herein we report a rare concurrency of bone leiomyosarcoma in a patient with a plexiform neurofibroma, adding to the few reported cases of leiomyosarcomas in patients with NF1. Our case is a 14-year-old male who is a known case of NF1 and presented with a four-month history of pain and swelling on the medial side of the right knee. Imaging and biopsy confirmed the diagnosis of leiomyosarcoma. Based on the authors' knowledge and search, this is the first reported case of plexiform neurofibroma with a primary bone leiomyosarcoma, representing an extremely rare concurrency. Patients with such uncommon tumors should be assessed regularly, and continuous follow-up is essential.

## Introduction

Neurofibromas are the most common benign peripheral nerve tumors infrequently present in patients with the autosomal dominant syndrome neurofibromatosis type 1 (NF1). NF1 affects roughly 1 in 3500 newborns and is considered one of the most commonly occurring genetic diseases [1]. Plexiform neurofibromas represent a rare variant (30%) of NF1 in which the nerve fibers thicken due to the spread of tumor cells along nerve fascicles [2]. Distinguishing features comprise Lisch nodules (iris hamartomas), pigmented skin lesions (e.g. cafe-au-lait spots), organ or tissue damage (visceral, skeletal, and neural), and malignant or benign tumors. A mutation on chromosome 17q11.2 causes NF1 by affecting the synthesis of neurofibromin (a tumor suppressor protein), which is expressed at high levels in the nervous system in unaffected individuals. Conversely, a low level of neurofibromin is associated with developing benign and malignant tumors, mainly of the nervous system, such as malignant peripheral nerve sheath tumors (MPNST), gliomas, and schwannomas [3,4].

Leiomyosarcoma is a malignant tumor that usually affects the soft tissues, such as the soft tissues of the extremities, uterus, and gastrointestinal system. Primary leiomyosarcoma of the bone is a rare tumor that affects mainly the metaphysis of long bones, particularly the proximal tibia and distal femur [2]. Furthermore, NF1 patients can develop leiomyosarcoma less frequently than other malignancies.

## Case presentation

**Figure 1:**
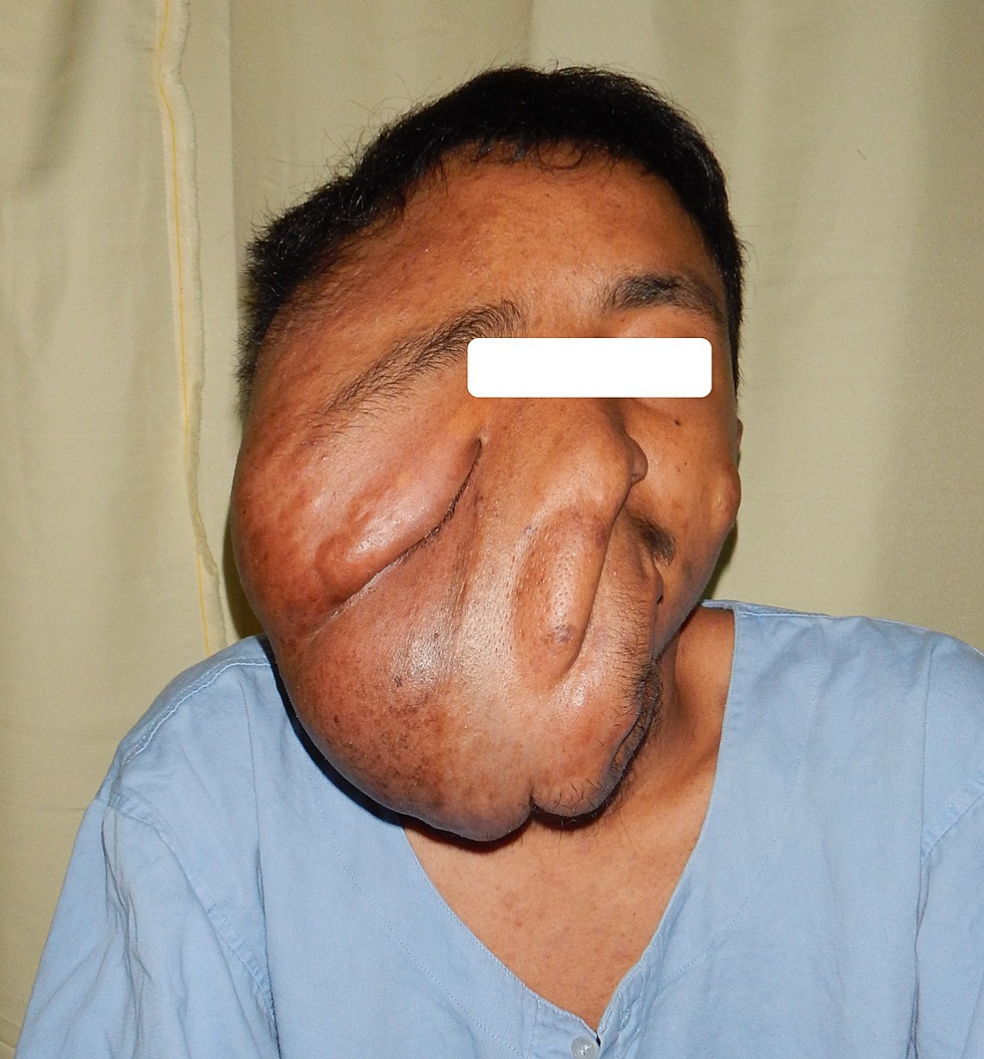
Photograph shows right hemi-facial plexiform neurofibroma.

**Figure 2:**
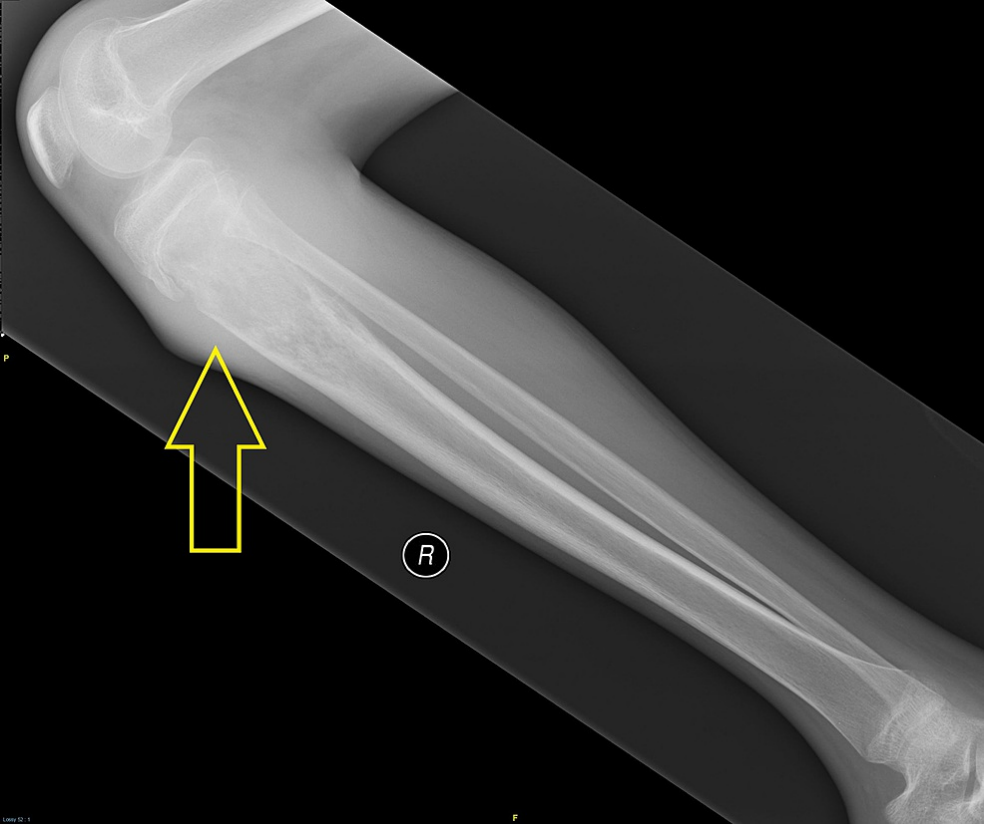
X-ray shows an ill-defined lytic lesion at the proximal third of the right tibia with periosteum elevation (yellow arrow).

A 14-year-old Malay male known case of NF1 (Figure 1) presented with a four-month history of pain and swelling on the medial side of the right knee, and a limping gait, preceded by a history of fall. Local examination showed an 8x5 cm ill-defined mass over the medial aspect of the right knee, which was hard and tender with restriction of knee joint movement. Radiographs of the right knee revealed an ill-defined lytic lesion at the proximal third of the right tibia with periosteum elevation (Figure 2). MRI of the right knee revealed an irregular lobulated enhancing mass measuring 4.2x4.4x6.9 cm involving the proximal metadiaphysis of the right tibia. The chest CT scan was normal. A bone scan revealed no evidence of skeletal metastasis.

**Figure 3:**
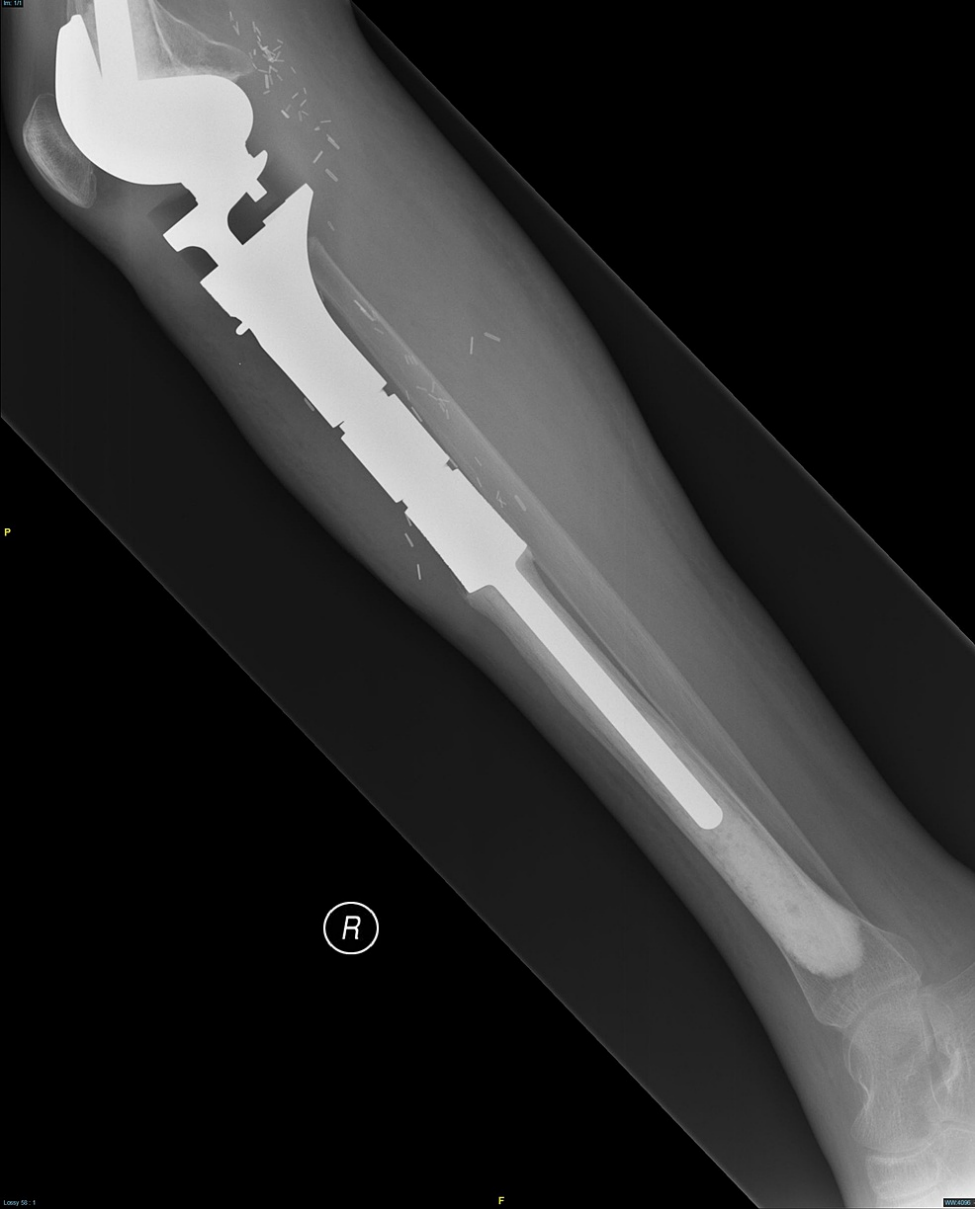
X-ray shows endoprosthesis in the right lower limb after resection of the tumor.

The tumor was resected with endoprosthesis insertion (Figure 3) and the wound covered with free latissimus dorsi flap. The patient completed 30 cycles of radiotherapy without complications. He is currently more than five years post-operative, ambulating unsupported, healthy, and has no evidence of tumor recurrence.

## Discussion

NF1 is an autosomal dominant disorder that decreases the synthesis or function of neurofibromin (tumor suppressor protein production), increasing the risk of benign and malignant soft tissue tumors [5]. One of the rare neurofibroma variants is plexiform neurofibromas, in which the spread of tumor cells along nerve fascicles leads to a diffuse mass of thickened nerve fibers, involving connective tissue and skin folds that appear as bags of worms. In about 10% of cases, these lesions progress to MPNST [5,6]. NF1 is more susceptible to developing benign and malignant neoplasms, mainly of neurogenic or neuroendocrine origin. When compared to the general population, people with NF1 have a four- to six-fold increase in the risk of developing a malignancy (24%), according to Zöller et al. [7] and recent research studies [4]. In addition, unusual tumors are more frequent in patients with NF1, such as pheochromocytoma, carcinoid tumor, chronic myeloid leukemia, brain tumors, and MPNST. Compared to the common tumors such as lung, breast, colon, kidney, and prostate cancer, all of which can occur, but their occurrence is less than in the normal population [2]. Most of the NF1 malignant transformations are progressed to MPNST, but fewer cases may be accompanying heterologous differentiation components. Common heterologous components are chondrosarcoma, rhabdomyosarcoma, angiosarcoma, and osteosarcoma [4].

In children and adolescents with NF1, soft-tissue sarcomas account for roughly 8% of malignant tumors [1]. Smooth muscle leiomyosarcomas account for 10-20% of all diagnosed sarcomas. Primary bone leiomyosarcoma is extremely rare, accounting for less than 0.1% of all primary bone tumors [8]. Primary bone leiomyosarcoma was never reported in NF1.

The different localizations of leiomyosarcomas reported in NF1 patients were intracranial, liver, hand, sciatic nerve, pelvis, and bladder [7,9]. Afşar et al. summarized NF1 patients developing rare tumors from 1989 until 2013. The results generated by the literature search summarized the clinical characteristics of 43 patients with NF1 and somatostatinoma, eight patients with osteosarcoma, and 15 patients with leiomyosarcomas, which were found at different locations, including the sciatic nerve, liver, bladder, and intestine [10]. Compared to Zöller et al. [7], they reported only two cases of leiomyosarcoma amongst 70 patients with NF1. A recent study conducted in 2020 by Landry et al. [9] on 1607 patients with NF1 found 666 (41.4%) patients who developed other neoplasms in addition to neurofibromas. Among the patients in the study group, 285 (17.7%) developed sarcomas, and only two (0.1%) developed leiomyosarcoma. This different localization illustrates the need to be aware of potential leiomyosarcoma in NF1. Many studies report and prove that wide local excisions of the mass, followed by radiation therapy, are the most accepted treatment method [5].

## Conclusions

This case can be added to the few reported cases in the literature describing leiomyosarcomas in patients with NF1. It raises the voice that examining the patients routinely and considering leiomyosarcoma in the differential diagnosis is crucial to getting a better outcome. It highlights that physicians should be perceptive that there is a possibility of malignant tumors in NF1 patients and their histological characteristics and prognosis. Understanding the different presentations and the early establishment of an accurate diagnosis can be advantageous in commencing timely management and reducing morbidity and mortality.
